# Effects of exogenous calcium on flavonoid biosynthesis and accumulation in peanut roots under salt stress through multi-omics

**DOI:** 10.3389/fnut.2024.1434170

**Published:** 2024-10-30

**Authors:** Yan Gao, Xuan Dong, Rongjin Wang, Yongyong Zhang, Fei Hao, Xuguang Niu, Hui Zhang, Guolin Lin

**Affiliations:** ^1^College of Land and Environment, Shenyang Agricultural University, Shenyang, Liaoning, China; ^2^National Engineering Research Center for Efficient Utilization of Soil and Fertilizer Resources, Shenyang, Liaoning, China; ^3^Panxi Crops Research and Utilization Key Laboratory of Sichuan Province, Xichang University of Sichuan Province, Xichang, Sichuan, China

**Keywords:** flavonoids, salt stress, exogenous calcium, peanut, UPLC-MS/MS

## Abstract

Flavonoids possess antioxidant properties and are crucial in enhancing plant resistance to abiotic stress. Exogenous calcium has been found to regulate the biosynthesis and accumulation of secondary metabolites, including flavonoids. However, the mechanism by which exogenous calcium influences flavonoid regulation in peanut roots under salt stress remains unclear. In this study, four treatment conditions were established: no salt stress, salt stress, exogenous calcium, and a combination of salt stress and exogenous calcium. The peanut root flavonoid profile was comprehensively analyzed using both a broadly targeted metabolomic approach and an absolute quantitative flavonoid metabolome. A total of 168 flavonoids were identified in the broad-target metabolome, while 68 were quantified in the absolute quantification analysis. The findings revealed that salt stress generally increased flavonoid content in peanut roots, while co-treatment with exogenous calcium significantly reduced this accumulation. Additionally, the activities of key enzymes and the expression of genes involved in the flavonoid biosynthesis pathway were upregulated under salt stress, but downregulated following the combined treatment. This study offers valuable insights into the physiological and ecological roles of flavonoids in response to environmental stressors in economically important crops.

## 1 Introduction

Flavonoids are constituents of the phenylpropanoid class of compounds, characterized by their fundamental structure of C6-C3-C6 (1). Furthermore, flavonoids serve myriad pivotal functions within plants, impacting not only growth and development but also acting as antioxidants amidst both biotic and abiotic stressors (2). Flavonoids, revered as a significant category of secondary metabolites, exhibit wide distribution across diverse plant tissues. Their accumulation within plants confers heightened resilience against abiotic stressors (3). Studies have demonstrated that flavonoids are pivotal in mitigating stress-induced damage within plants. They achieve this by curbing the accumulation of ROS or enhancing stress tolerance through synergistic interactions with other stress response factors, such as plant hormones (4). The process of flavonoid biosynthesis initiates with the phenylalanine pathway and encompasses a cascade of enzymatic reactions occurring within the endoplasmic reticulum (1). Based on the extent of oxidation within the heterocyclic ring and the presence of hydroxyl or methyl groups on the benzene ring, flavonoids can be classified into twelve distinct subclasses. These include but are not limited to, flavones, flavonols, isoflavones, chalcones, flavanones, flavanols, and anthocyanins (5). These metabolites are usually produced by 4-coumaroyl-CoA and are mainly catalyzed by chalcone isomerase (CHI), flavonol synthetase (FLS), flavone synthase (FNS), flavanone 3-hydroxylase (F3H), flavonoid 3′-hydroxylase (F3’H), UDP-glycosyltransferase (UGT) and isoflavone synthase (IFS) (6). Studies have reported that the dynamic fluctuations in flavonoid levels exhibit a significant capacity to alleviate the deleterious effects of saline-alkali stress on the growth and developmental processes of sorghum (1). However, as far as our knowledge extends, the potential role of flavonoids in mitigating salt stress, particularly in conjunction with exogenous calcium, remains unexplored within peanut roots.

Soil salinization poses a pressing global challenge, persistently escalating in severity (7). Furthermore, it stands as one of the paramount and prevalent environmental issues in arid and semi-arid regions across the globe (8, 9), the global extent of salinized land has surged to approximately 1 billion hectares, encompassing around 7% of the total landmass (10). In addition, secondary salinization has reached 7,700 hectares (11, 12). It significantly impedes agricultural production and industry development, reducing soil fertility and productivity and decreasing crop yields (13). Hence, there is a pressing need to enhance salinised soil management and utilization strategies (14). It stands as a pivotal challenge demanding resolution. Nonetheless, certain plant species have evolved an array of mechanisms to thrive in salinized soils, manifesting across various levels such as signal transduction, the regulation of gene expression, protein synthesis and turnover, and carbohydrate and energy metabolism (15, 16).

Calcium (Ca) is a fundamental nutrient element for facilitating plant growth and development. It serves as a secondary messenger, intricately regulating many metabolic processes, ranging from cell division and apoptosis to establishing cell polarity, differentiation, and senescence (17, 18). Furthermore, calcium participates in numerous vital cellular processes, including preserving cell membrane and cell wall integrity, augmenting the activity levels of myriad energy enzymes, and modulating interactions with plant hormones (19). Calcium additionally alleviates detrimental stress impacts by modulating antioxidant metabolism (20). Research findings indicate that applying exogenous calcium enhances salt stress tolerance in bread wheat by regulating antioxidant mechanisms (21). Calcium ions impede sodium ions’ absorption, thereby ameliorating salt ions’ impact on plants. Moreover, calcium ions curtail the outflow of potassium ions from plant cells, mitigating the detrimental consequences of sodium chloride on plant growth (22). Hence, employing calcium as an exogenous treatment across various plant species is imperative for enhancing plant tolerance to salt stress.

Peanut (*Arachis hypogaea* L.) is a prominent oilseed crop cultivated globally, primarily for its economic significance. China, one of the leading producers and exporters of peanuts worldwide, extensively cultivates this versatile crop (23, 24). Simultaneously, peanuts serve as a crucial oil crop and protein source for human consumption, annually contributing to 20% of global oil production and supplying 11% of the world’s protein needs (25). Furthermore, most peanuts cultivated worldwide have extensive applications in various products, including peanut butter, candies, roasted snacks, and filler ingredients in meat dishes, soups, and desserts, aside from being used for oil extraction (26). It is well established that peanuts contain various bioactive compounds, including polyphenols and flavonoids (3). However, research on flavonoid accumulation and biosynthesis in peanuts remains limited, with few studies exploring the mechanisms behind the induction of monomeric flavonoids in peanut roots under salt stress and exogenous calcium treatment.

In previous studies, the analysis of peanut metabolites primarily focused on a limited number of stilbenoids, such as resveratrol, without conducting a comprehensive and precise screening of flavonoids in peanut root systems. We hypothesize that salt stress induces the production of secondary metabolites, including flavonoids, in the plant’s roots, while exogenous calcium decreases flavonoid biosynthesis under salt stress, thereby mitigating its adverse effects. Our earlier research demonstrated that exogenous calcium helps regulate the balance between enzymatic (e.g., antioxidant enzymes) and non-enzymatic systems (e.g., flavonoids) in peanut roots under salt stress, providing relief. This study aims to explore the impact of exogenous calcium on flavonoid content in peanut roots during salt stress and to uncover the underlying mechanisms by examining metabolites, enzymes, and gene expression. Additionally, we tracked the levels of five monomeric flavonoids at multiple time points, while also analyzing changes in total flavonoids, total phenolics, and antioxidant capacity throughout the treatment. Our findings offer new insights into the dynamic shifts and molecular mechanisms of salt stress and exogenous calcium on flavonoids in peanut roots. This information enhances our ability to harness the potential of flavonoids and furthers our understanding of how exogenous calcium alleviates salt stress in peanut root systems.

## 2 Materials and methods

### 2.1 Materials

The peanut seeds “haihua 1” were harvested in late October 2022 and sourced from Qilu Seed Mall. Upon arrival at the laboratory, the samples were promptly refrigerated at-4°C for storage. Rutin, hyperoside, daidzein, liquiritigenin, and formononetin (all of UPLC grade) were procured from Yuanye Biotechnology Co., Ltd. (Shanghai, China) for subsequent UPLC analysis.

### 2.2 Conditions for germination and cultivation of peanuts

Peanut seeds with intact seed coats were randomly selected and sterilized using a 1% sodium hypochlorite solution for 3 min, followed by thorough rinsing with deionized water on three occasions. Subsequently, the sterilized peanut seeds were soaked in deionized water at 25°C for 10 h. Upon completing the soaking process, the peanut seeds were carefully transferred to trays lined with moist towels and placed in an environment with a temperature of 28°C and relative humidity of 80% for 48 h to facilitate germination. The sprouted, uniformly sized young shoots were carefully selected and transferred to porous plastic culture pots designed for peanuts, each measuring 15.5 cm in width and 16 cm in height. The pots were then placed in an incubator and kept under controlled conditions for 3 days at 25°C, 80% relative humidity, and in complete darkness. Afterward, peanut seedlings with root lengths 1.5 times that of the embryonic axis were selected for treatment with the prepared solution for 72 h. The treatment solution volume was set at 3.1 L, and the concentrations of NaCl and CaCl_2_ were determined based on pre-experimental concentration screening conducted by the laboratory team. Four different treatment groups were established in this study: (1) CK (distilled water); (2) Na treatment (150 mM NaCl); (3) Ca treatment (15 mM CaCl_2_); and (4) Na_Ca treatment (150 mM NaCl and 15 mM CaCl_2_). Each culture pot accommodated fifty peanut seedlings, with three biological replicates established for every treatment. Peanut root samples were collected at six distinct time intervals during germination: 12 h, 24 h, 36 h, 48 h, 60 h, and 72 h, respectively. Upon collection, root samples were swiftly frozen using liquid nitrogen and subsequently stored in a refrigerator set to-80°C for subsequent determination of relevant parameters.

### 2.3 Widely targeted metabolomic detection

Based on the results of our previous pre-experiment, we identified 48 h post-treatment as one of the optimal time points for exogenous calcium to mitigate salt stress. Therefore, peanut root samples from this time point were collected for omics testing. The widely targeted metabolome of peanut roots was meticulously extracted and identified by Metware Biotechnology Co., Ltd. (Wuhan, China). Following extraction, the biological samples underwent vacuum drying using a freeze dryer (Scientz-100F, Zhejiang, China). They were ground into a fine powder form using a ball mill (MM 400, Retsch, Germany) operating at 30 Hz for 1.5 min. Next, 100 mg of powdered sample was dissolved in 1.2 mL of 70% methanol extract (V/V), and the resulting solution was thoroughly mixed using a vortex oscillator (MIX-200, Shanghai, China). After mixing, the samples underwent centrifugation at 12000 rpm for 10 min at 4°C, following which the supernatant was carefully aspirated. Subsequently, samples were filtered through microporous filter membranes with a pore size of 0.22 μm and stored in injection bottles for UPLC-MS/MS analysis.

Each group of samples underwent independent analysis with three biological replicates. The data acquisition system primarily comprised UPLC (SHIMADZU Nexera X2) coupled with Tandem mass spectrometry (Applied Biosystems 4,500 QTRAP). The liquid phase conditions predominantly featured Agilent SB-C18 (1.8 μm, 2.1 mm × 100 mm), with the solvent system comprising ultrapure water (0.1% formic acid, referred to as solvent A) and acetonitrile (0.1% formic acid, referred to as solvent B). The elution gradient commenced at 95:5 for A/B at 0 min, transitioned to 5:95 for A/B at 9 min, reverted to 95:5 for A/B at 10–11 min, and maintained 95:5 for A/B at 14 min. The flow rate was set at 0.35 mL/min, the column temperature maintained at 40°C, and the injection volume set at 4 μL.

The mass spectrometry conditions primarily encompassed LIT and triple quadrupole (QQQ) scans conducted on a state-of-art triple quadrupole linear ion trap mass spectrometer (Q TRAP), specifically the AB4500 Q TRAP UPLC-MS/MS system equipped with an ESI Turbo ion spray interface. This system is adeptly controlled by the Analyst 1.6.3 software (AB Sciex), allowing operation in positive and negative ionization modes. The ESI source was configured with a source temperature of 550°C, an ion spray voltage of 5,500 V (in positive ion mode) and-4500 V (in negative ion mode), while the ion source gas I, gas II, and curtain gas were set to 50, 60, and 25 psi, respectively. The QQQ scans were executed in MRM mode, with DP and CE parameters meticulously calibrated for each MRM ion pair. Tailored to the eluted metabolites in each period, a distinct set of MRM ion pairs was diligently monitored.

### 2.4 Absolute quantitative metabolome detection of flavonoids

Flavonoid metabolites underwent extraction and identification procedures conducted by Metware Biotechnology Co., Ltd. (Wuhan, China). The pretreatment protocol for peanut roots mirrored the widely targeted metabolome extraction. UPLC was conducted utilizing a QTRAP6500+ instrument from SCIEX, coupled with a Waters ACQUITY UPLC HSS T3 C18 column (1.8 μm, 100 mm × 2.1 mm i.d.). While the detection method deviated slightly from the widely targeted metabolome detection, the mobile phase consisted of ultrapure water with 0.05% formic acid (solvent A) and acetonitrile with 0.05% formic acid (solvent B). The elution gradients were meticulously programmed: 90:10 for 0 min A/B, 80:20 for 1 min A/B, 30:70 for 9 min A/B, 5:95 for 12.5 min A/B, 5:95 for 13.5 min A/B, 90:10 for 13.6 min A/B, and 90:10 (V/V) for 15 min A/B. The column temperature was set at 40°C, the flow rate was maintained at 0.35 mL/min, and the injection volume was set at 2 μL.

For tandem mass spectrometry analysis, the SCIEX 6500+ QTRAP was utilized. The instrument conducted linear ion trap (LIT) and triple quadrupole (QQQ) scans within a SCIEX 6500+ QTRAP UPLC-MS/MS system equipped with an ESI turbo ion spray interface. The ESI source operates under specific conditions: the electrospray ion source temperature is maintained at 550°C, the ion spray voltage (IS) is set at 5500 V for positive ion mode and-4500 V for negative ion mode, while the curtain gas (CUR) pressure is regulated at 35 psi. Within the Q-Trap 6,500+, each ion pair is meticulously scanned based on optimized decluster voltage and collision energy parameters.

### 2.5 Determination of monomer flavonoid content

This study identified five individual monomeric flavonoids in peanut roots using UPLC-MS/MS analysis, including rutin, hyperoside, daidzein, liquiritigenin and formononetin. The extraction and determination methods were the same as the absolute quantitative metabolomics of flavonoids determined by Metware Biotechnology Co., Ltd. Specific information is in Supplementary Table S3.

### 2.6 Assessment of total phenolics, total flavonoids, and antioxidant capacity

Antioxidant capacity was evaluated through two distinct methods: DPPH free radical scavenging activity and ABTS free radical scavenging activity. Sample extraction, along with the determination of total phenols, total flavonoids, and antioxidant capacity, was performed following the methods outlined by Wu (27) and Zhu (3), with minor modifications applied to the sample extraction procedures. Their units were denoted as gallic acid equivalent (mg GAE/g FW), catechin equivalent (mg CET/g FW), Vitamin C equivalent (μg Vc/g FW), and Trolox equivalent (μg Trolox/g FW), respectively.

### 2.7 Assessment of key enzyme activities within the flavonoid biosynthesis pathway

CHI, FLS, FNS, F3H, F3’H, UGT, and IFS activities were determined using an enzyme-linked immunosorbent assay (ELISA). Samples were extracted from peanut roots cultivated under identical conditions. Each treatment comprised three biological replicates, each consisting of mixed root samples from at least five plants. The enzyme above activities was assessed in peanut root samples using commercially available Plant ELISA Kits (BL5055-A, BL4996-A, BL50621-A, BL7952-A, BL50706-A, BL8093-A, BL50646-A, respectively; Jiangsu Baolai Biotechnology Co., Ltd., China), following the manufacturer’s instructions.

### 2.8 Real-time quantification polymerase chain reaction

Total RNA extraction and purification were conducted utilizing the RNAiso Pure Plant Kit (Takara, Japan). The quantity and quality of RNA were assessed using an ultramicro UV spectrophotometer (IMPLEN-N50, Thermo Scientific, United States) and 2% agarose gel electrophoresis (D50-UVIPURE, UVITE, United Kingdom; EPS300, Tanon, China). Subsequently, RNA was reverse transcribed into cDNA for subsequent RT-qPCR analysis employing the GoScriptTM Reverse Transcription kit (Promega, United States). Relative quantification methods were employed for data analysis (28) with primer design facilitated by Primer 5.0 software. The peanut Actin-7 (LOC112715878) gene served as the internal control. The results were calculated using the 2-ΔΔCt method, all RT-qPCR expression data were derived from three biological samples analyzed in triplicate. Details of the primers utilized for RT-qPCR can be found in Supplementary Table S4.

### 2.9 Statistical analyses

The statistical analysis of all data was conducted using IBM SPSS 22.0 statistical software (SPSS Inc., Chicago, IL, United States). Differences among means were assessed using Duncan’s multiple range test, with statistical significance set at a *p* value of <0.05. Data visualization was accomplished using OriginPro 2021 (OriginLab, Northampton, MA, United States).

## 3 Results

### 3.1 Identification and classification of metabolites and flavonoids in peanut roots

**Figure 1:**
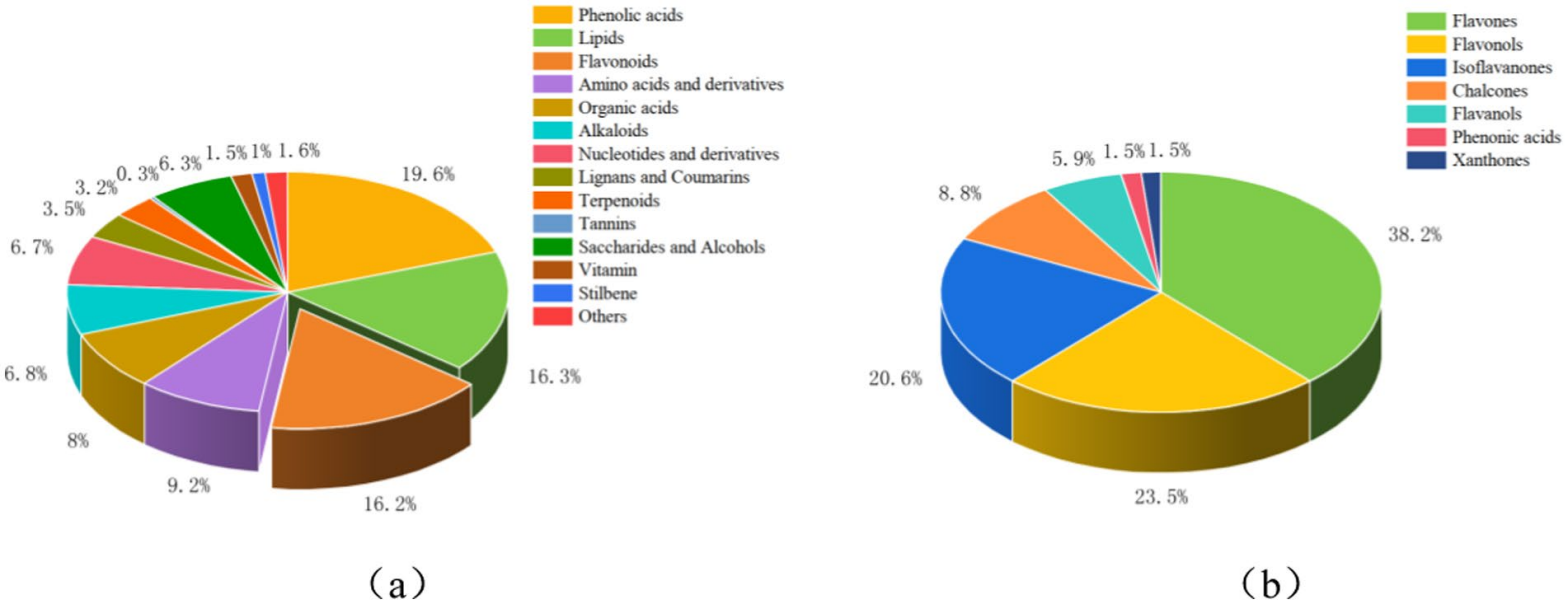
Types and proportions of metabolites in the peanut root system. (a) Species and proportions of 1,037 primary and secondary metabolites in the peanut root system. (b) Types and proportions of 68 flavonoids in the peanut root system. Figure (a) shows the data measured for the broadly targeted metabolome. (b) Shows the data measured for the absolute quantitative metabolome of flavonoids.

To gain a deeper understanding of how exogenous calcium regulates metabolites in the root system of peanut seedlings under salt stress, we conducted a comprehensive analysis using a broadly targeted metabolomics approach. 1,037 metabolites were successfully identified (Figure 1a; Supplementary Table S1), spanning 14 distinct classes. These include Phenolic acids (19.6%), Lipids (16.3%), Flavonoids (16.2%), Amino acids and derivatives (9.2%), Organic Acids (8%), Alkaloids (6.8%), Nucleotides and derivatives (6.7%), Lignans and Coumarins (3.5%), Terpenoids (3.2%), Tannins (0.3%), Saccharides and Alcohols (6.3%), Vitamin (1.5%), Stilbene (1%), and Others (1.6%). Phenolic acids, Lipids, and Flavonoids emerged as the predominant metabolite classes. Flavonoids are also known to be phenolic compounds, so we further performed absolute quantitative metabolomics detection of flavonoids in peanut roots 48 h after treatment. A comprehensive total of 68 flavonoids were successfully identified (Figure 1b; Supplementary Table S2), further classified into 26 flavones, 16 flavonols, 14 isoflavones, 6 chalcones, 4 flavonols, 1 phenolic acid, and 1 xanthone. Overall, Na treatment increased flavonoid species and accumulation compared to CK treatment. The Ca treatment decreased the amount of flavonoid accumulation and the number of species compared to the Na_Ca treatment.

### 3.2 Changes of five monomer flavonoids content in peanut roots

**Figure 2:**
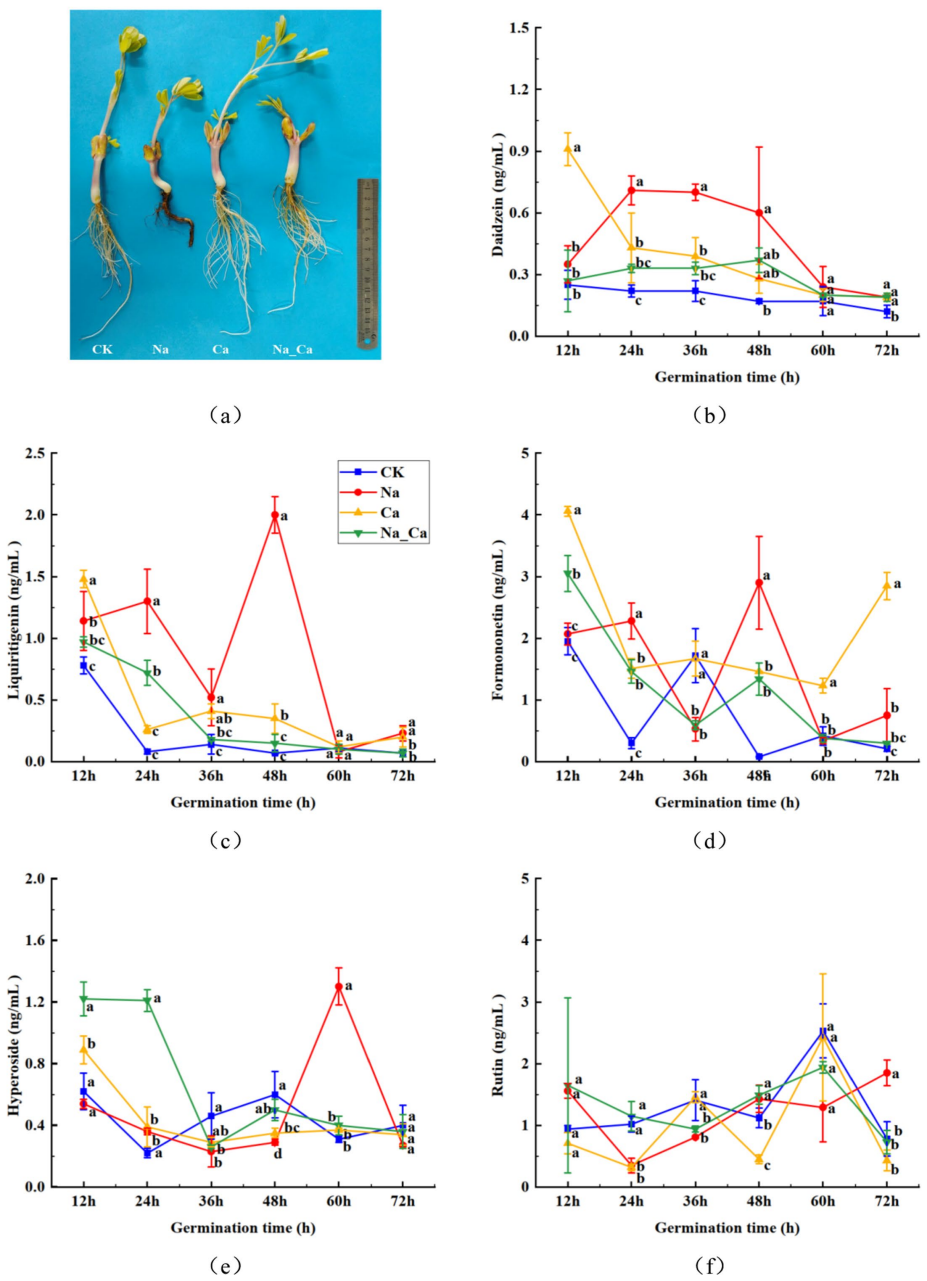
Changes of flavonoid contents in peanut roots of different treatments. (a) Phenotype of peanuts roots under different treatments. (b) Daidzein. (c) Liquiritigenin. (d) Formononetin. (e) Hyperoside. (f) Rutin.

The roots of peanut seedlings under different treatments showed significant differences, with root growth significantly inhibited under salt stress, while the addition of exogenous calcium to the salt stress treatment significantly promoted root growth (Figure 2a). To delve deeper into the dynamics of flavonoids within the roots across various treatments during peanut germination, we selected five monomeric flavonoids from flavonoids, flavonols, and isoflavonoids for serial determination at six time points (Figures 2b–f). The concentration of daidzein within peanut roots exhibited a declining trajectory (Figure 2b). Notably, daidzein peaked at 0.91 ng/mL FW under Ca treatment after 12 h, only gradually diminishing during the ensuing incubation period. Conversely, daidzein levels remained relatively stable in the control group, exhibiting marginal fluctuations throughout the incubation process. Between 24 and 36 h, the daidzein accumulation in peanut roots subjected to Na_Ca treatment markedly lagged behind that of Na-treated counterparts. The concentration of liquiritigenin within peanut roots treated with NaCl exhibited a gradual decline over the initial 2 days of incubation, followed by a sharp surge to its peak value (2.00 ng/mL), before undergoing a subsequent rapid decrease to its minimum level (0.23 ng/mL) during the subsequent incubation period. Conversely, the other treatment groups demonstrated a steady downward trend (Figure 2c). The concentration of formononetin exhibited a gradual decline throughout the incubation period (Figure 2d). In peanut roots subjected to Na treatment, the formononetin content steadily decreased from 12 h to 36 h, spiked to its peak level (2.90 ng/mL) at 48 h, and subsequently experienced a sharp decrease during the ensuing incubation period. Between 60 h and 72 h, this content was notably higher in Ca treatment group, surpassing that of the other treatments. Between 12 h and 48 h, the presence of hyperoside in peanut roots subjected to Na treatment exhibited a gradual decline, followed by a sudden surge to its peak value (1.3 ng/mL FW), before sharply declining once more. Conversely, the three groups displayed a consistent downward trend (Figure 2e). The concentration of rutin in peanut roots exposed to NaCl rapidly declined from 12 h to 24 h, followed by a gradual ascent to its peak value (1.85 ng/mL) during the ensuing incubation period. Conversely, in the other three groups, rutin levels steadily rose to their maximum levels from 12 h to 60 h before experiencing a sharp decrease (Figure 2f).

### 3.3 Alterations in total phenolics, total flavonoids, and antioxidant capacity

**Figure 3:**
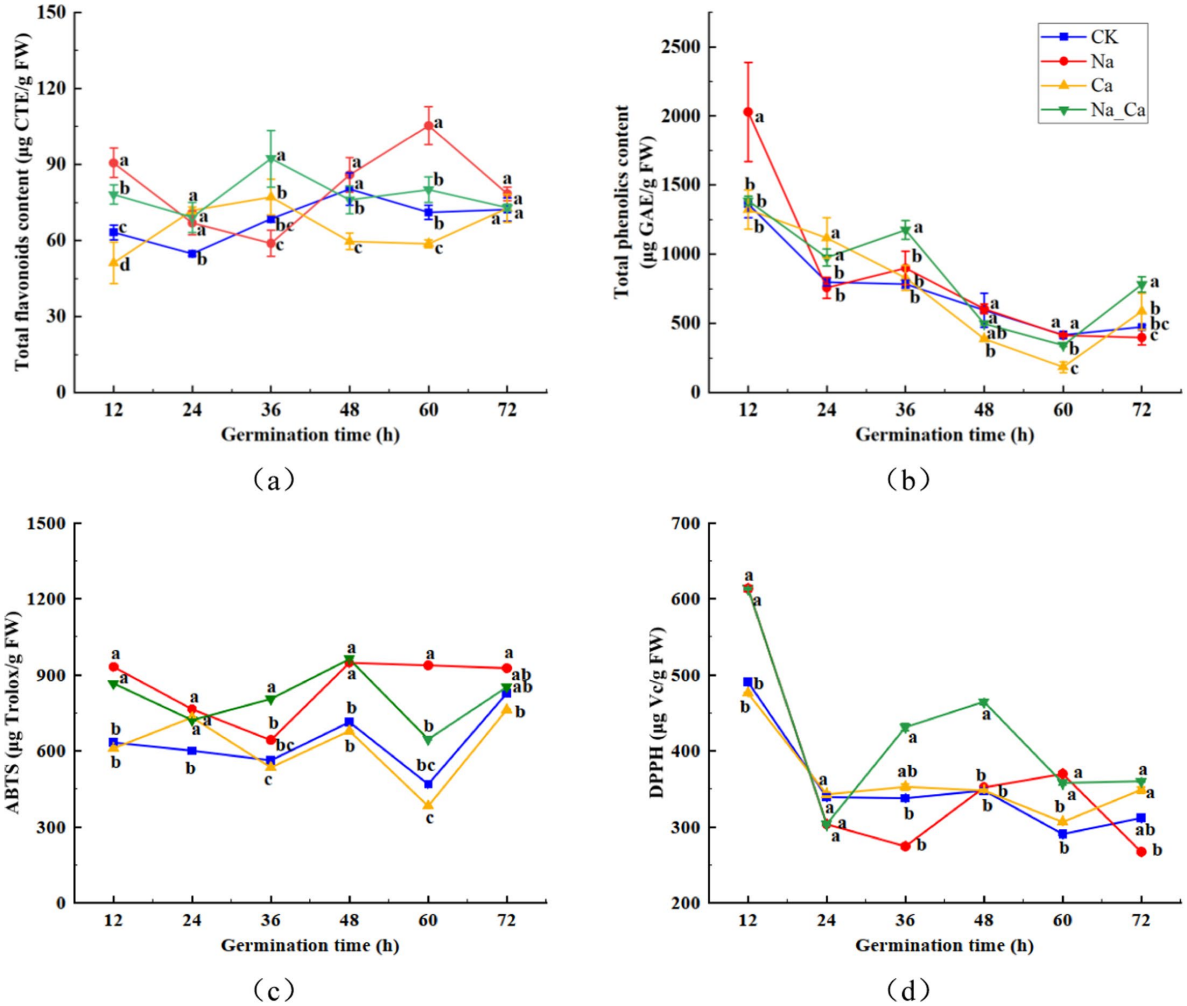
Changes of total flavonoids (a), total phenolics (b), ABTS (c), and DPPH (d) in peanut roots of different treatments. Different lowercase letters indicate a significant difference between different treatments at a time point (*p* < 0.05).

During the treatment period, the total flavonoid content in peanut roots under salt stress exhibited a pattern of initial decrease followed by an increase. In contrast, the other three treatments showed a trend of increasing first, then declining. The total flavonoid content peaked at 36 h for Ca and Na_Ca treatments, reaching 77.10 μg/g and 92.26 μg/g, respectively, whereas for CK treatment and Na treatment, it peaked at 48 h and 60 h, respectively, at 80.24 μg/g and 105.29 μg/g (Figure 3a). Conversely, the total phenol content gradually decreased over the treatment period. Notably, at 12 h of incubation, the total phenol content of Na treatment surpassed that of the remaining three groups. Moreover, the total phenol content of Ca and Na_Ca treatments increased from 60 h to 72 h (Figure 3b). Compared to the CK treatment, Ca treatment significantly reduced the ABTS scavenging capacity in peanut seedling roots, except at 24 h. In contrast, Na treatment significantly enhanced the ABTS scavenging capacity of the roots. When compared to Na treatment, Na_Ca treatment significantly lowered the ABTS scavenging capacity, except at 36 and 48 h, with reductions of 7.07, 5.63, 31.06, and 8.07% at 12, 24, 60, and 72 h, respectively (Figure 3c). The DPPH scavenging capacity of peanut roots decreased gradually during incubation. This capacity sharply declined across all groups from 12 h to 24 h, followed by a gradual decrease during the subsequent incubation. Notably, the DPPH scavenging capacity of Na_Ca treatment surpassed that of the remaining three groups from 36 h to 48 h (Figure 3d).

### 3.4 Alterations in key enzymatic pathways involved in flavonoid biosynthesis

**Figure 4:**
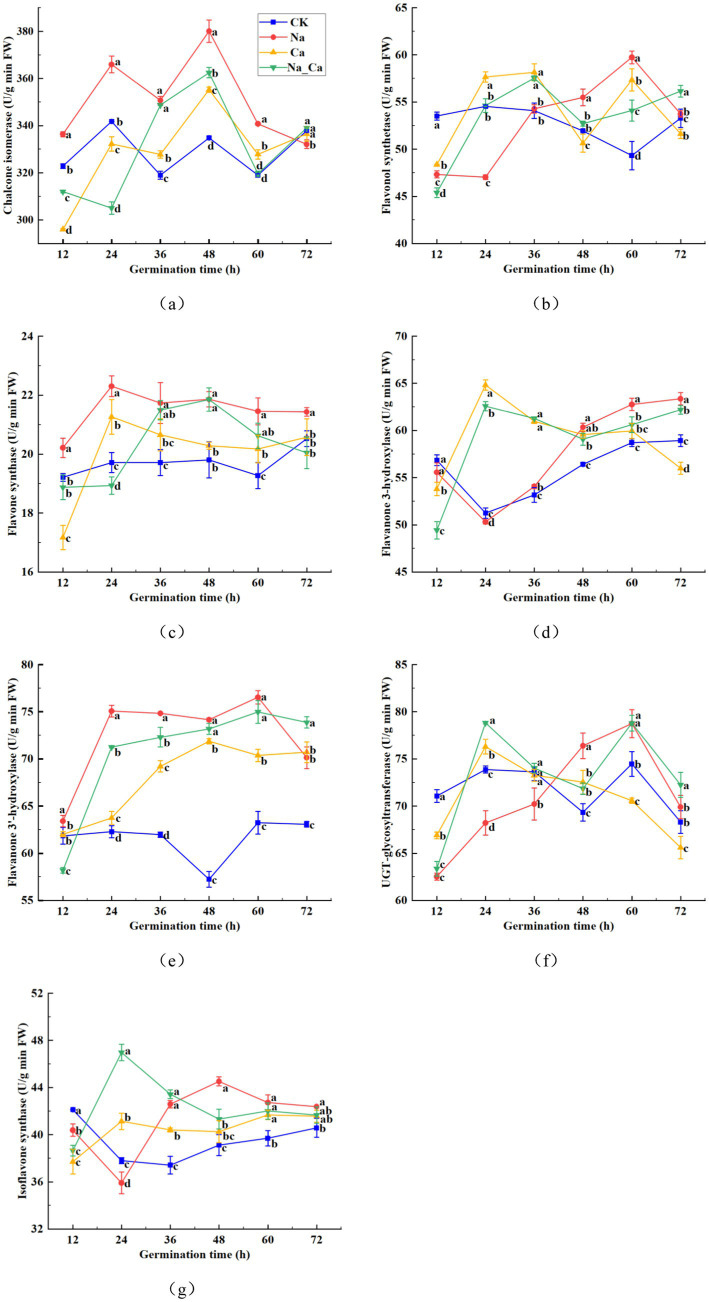
Changes of seven enzymes in the flavonoid biosynthetic pathway in peanut roots of different treatments. (a) Chalcone isomerase, CHI. (b) Flavonol synthetase, FLS. (c) Flavone synthase, FNS. (d) Flavanone 3-hydroxylase, F3H. (e) Flavonoid 3′-hydroxylase, F3’H. (f) UDP-Glycosyltransferase, UGT. (g) Isoflavone synthase, IFS. Different lowercase letters indicate a significant difference between different treatments at a time point (*p* < 0.05).

Supplementary Figure S1 illustrates the intricate plant flavonoid metabolic pathway. To delve deeper into the fluctuations of pivotal enzymes within the flavonoid biosynthesis pathway, we selected seven enzymes from the latter part of the biosynthesis pathway for measurement at six consecutive time points (Figure 4). The CHI activity in CK treatments fluctuated initially but eventually stabilized within the range of 320–340 U/g between 12 and 72 h. In comparison, Ca treatments significantly enhanced root CHI activity in peanut seedlings at 36, 48, and 60 h, while Na treatments significantly boosted CHI activity from 12 to 60 h, followed by a 1.71% reduction at 72 h. When compared to Na treatment, Na_Ca treatment significantly decreased CHI activity at 72 h, though no significant difference was observed at 36 h (Figure 4a). Similarly, FLS activity in all groups displayed an initial rise followed by a decline during the incubation period. Conversely, the FLS activity of the control group decreased during the first 60 h before rising again in the subsequent incubation period. From 48 h to 60 h, salt stress induced a significant increase in FLS activity in peanut roots (Figure 4b). In CK treatment, the FNS activity of peanut roots exhibited a steady increase, whereas in the other three groups, there was an initial rise followed by a subsequent decline. The FNS activity in CK-treated peanut seedlings fluctuated between 19.21 and 20.52 U/g throughout the treatment period. In comparison, Ca treatment significantly increased FNS activity in the root system from 24 to 72 h, though no significant difference was observed at 72 h. Salt stress treatments, however, markedly increased FNS activity from 12 to 72 h in both cases. Notably, Na_Ca treatment significantly reduced FNS activity in the peanut root system compared to Na treatment throughout the entire period (Figure 4c). These results suggest that exogenous calcium may help mitigate the effects of salt stress. The F3H activity in CK and Na treatments exhibited a gradual decline from 12 h, reaching a minimum, followed by a gradual increase. In contrast, Ca and Na_Ca treatments showed a sharp rise to a peak at 12 h, then gradually decreased. Compared to CK treatment, Na treatment significantly increased F3H activity in the peanut root system by 1.67, 6.97, 6.92, and 7.55% at 36, 48, 60, and 72 h, respectively. However, Na_Ca treatment significantly reduced F3H activity at 48, 60, and 72 h compared to Na treatment. This suggests that exogenous calcium helps mitigate the effects of salt stress on the peanut root system in the later stages of treatment (Figure 4d). F3’H activity in peanut roots exhibited an upward trajectory across all three treatment groups except CK treatment (Figure 4e). Under CK treatment, F3’H activity displayed minimal fluctuation, declining after 36 h and gradually rebounding during subsequent incubation. Notably, Na treatment, Ca treatment, and Na_Ca treatment substantially boosted F3’H activity, except at the 12 h mark. Conversely, the overall trend of UGT activity in peanut roots slightly decreased under CK and Ca treatments, whereas it showed an increasing pattern under Na and Na_Ca treatments. UGT activity observed in Na treatment significantly surpassed that of Na_Ca treatment at the 48 h mark (Figure 4f). The IFS activity in the roots of CK-treated peanut seedlings showed a gradual decline to a minimum of 37.41 U/g, followed by a gradual increase. In comparison, Ca treatment significantly enhanced IFS activity in peanut roots from 24 to 72 h, while Na treatment led to a significant increase starting at 36 h, peaking at 13.81% higher at 48 h. Compared to Na treatment, Na_Ca treatment significantly reduced IFS activity in peanut roots from 48 to 72 h, although no significant difference was observed at 60 and 72 h (Figure 4g).

### 3.5 Transcriptional expression of key enzyme genes in flavonoid biosynthesis

**Figure 5:**
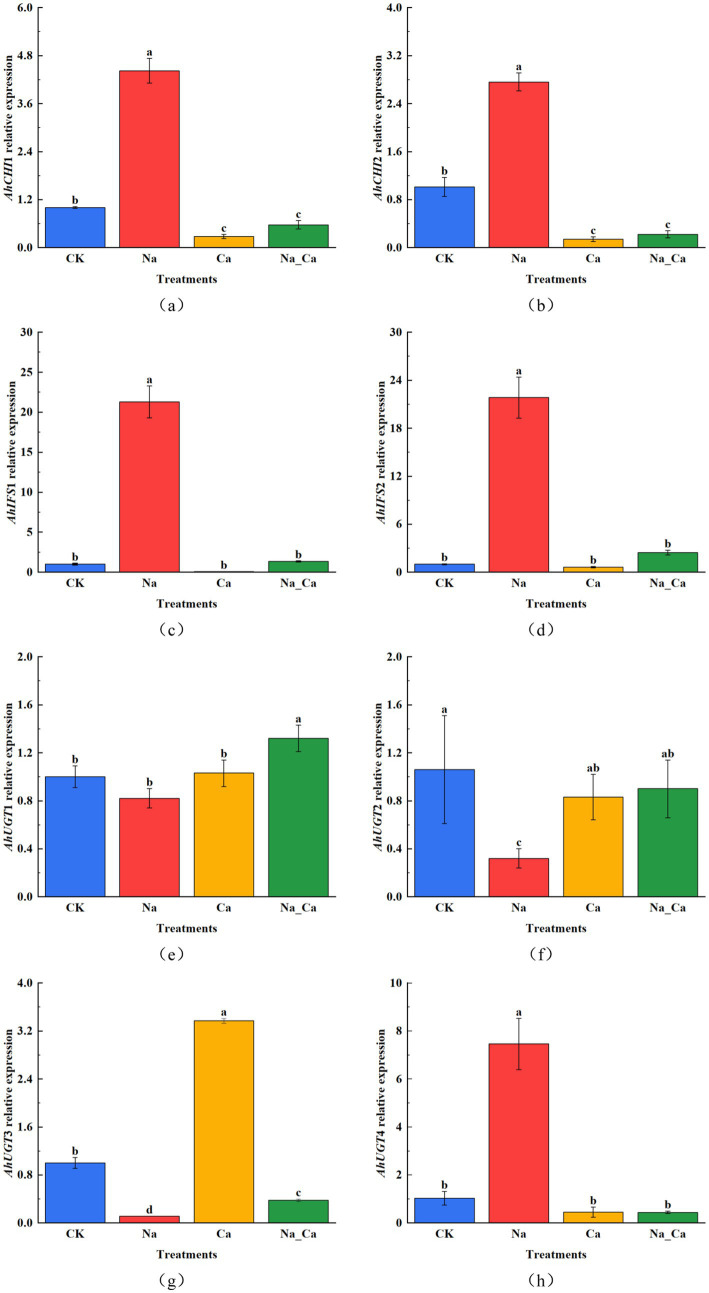
The changes of key enzyme genes in flavonoid biosynthesis at 48 h under different treatments. (a) *AhCHI1*, (b) *AhCHI2*, (c) *AhIFS1*, (d) *AhIFS2*, (e) *AhUGT1*, (f) *AhUGT2*, (g) *AhUGT3*, and (h) *AhUGT4*. Different lowercase letters indicate a significant difference between different treatments at a time point (*p* < 0.05).

To comprehend the variations in key enzyme genes within the flavonoid biosynthesis pathway across four distinct treatments, we assessed the expression of eight selected genes using qRT-PCR (Figure 5). *AhCHI1* and *AhCHI2* represent two distinct genes responsible for encoding CHI. *AhCHI1* and *AhCHI2* exhibited a consistent pattern across the four treatments (Figures 5a,b). Specifically, the relative expression levels of these genes peaked under Na treatments, showcasing a 4.42-fold and 2.76-fold increase compared to control, respectively. Conversely, Ca treatment elicited a down-regulation in the transcription of these genes relative to control. In stark contrast, Na_Ca treatment significantly up-regulated both *AhCHI1* and *AhCHI2* in comparison to Ca treatment. *AhIFS1* and *AhIFS2* represent two genes responsible for encoding IFS. Interestingly, under Ca treatment, there was a notable decrease in the expression of *AhIFS1* and *AhIFS2* in peanut roots compared to control. Conversely, Na treatment led to a significant increase in the relative expression of these genes. Notably, the changes in the abundance of *AhIFS1* and *AhIFS2* transcripts observed in Na_Ca treatments fell between those observed in Na treatment and Ca treatment (Figures 5c,d). *AhUGT1*, *AhUGT2*, *AhUGT3*, and *AhUGT4* correspond to four UGT genes. Notably, the relative expression patterns of *AhUGT1* and *AhUGT2* exhibited similarities (Figures 5e,f). Both Na treatments resulted in a significant down-regulation of the expression of these two genes in peanut roots compared to CK treatments. Interestingly, *AhUGT1* displayed the highest expression in Na_Ca treatments, surpassing that of the control by 1.32-fold. In contrast, *AhUGT2* exhibited its peak expression in CK treatment, with Na treatment significantly reducing its expression in peanut roots. Unlike the response observed in Na treatment, the expression of *AhUGT2* was notably down-regulated in the Ca and Na_Ca treatment groups. The expression patterns of the two UGT genes, *AhUGT3* and *AhUGT4*, exhibited stark contrasts under Na and Ca treatments (Figures 5g,h). *AhUGT3* demonstrated its lowest relative expression under Na treatment, while it exhibited the highest expression level in Ca treatment (2.37-fold higher than the control). Conversely, *AhUGT4* displayed the highest relative expression under Na treatment (6.46-fold higher than control) and the lowest under Ca treatment. The four UGT genes showcased distinct patterns of change across the four treatments, suggesting their membership in a multi-gene family with diverse functions in flavonoid biosynthesis.

## 4 Discussion

In this study, we delved into the mechanism underlying the impact of exogenous calcium on flavonoids in peanut roots under salt stress across three key levels: flavonoids, enzymes, and gene transcription. This research represents the most extensive investigation into determining primary and secondary metabolites in peanut roots under salt stress, surpassing previous studies in scope and depth. Salt stress has emerged as a prominent environmental challenge, curtailing seed germination, seedling growth, and crop yield. Signalling molecules such as calcium serve as pivotal second messengers, mitigating the detrimental effects of salt stress across various crop species (21). We also observed phenolic acid as the most abundant secondary metabolite in peanut roots, and flavonoids ranked third after lipids. We hypothesize that this phenomenon could be attributed to salt stress triggering the plant’s inherent resilience against external adversities. Consequently, it prompts the production of an increased array of secondary metabolites rich in phenolic hydroxyl groups, responding to the secondary oxidative stress induced by salt. This mechanism aids in scavenging excessive ROS, sustaining plant growth and development amidst challenging conditions (29). This aligns with findings reported in oilseed rape. The leaves of oilseed rape treated with NaCl produced more differentially expressed metabolites, especially carboxylic acids and their derivatives and flavonoids (30). Flavonoids are renowned for their remarkable antioxidant properties, which mitigate plant stress by inhibiting the accumulation of ROS or by enhancing stress tolerance in collaboration with other stress response mechanisms (4). It can be observed from the absolute quantitative metabolome of flavonoids, flavones, flavonols, and isoflavones account for a higher proportion of the three subcategories of flavonoids (31–33). A recent study on sorghum had a result that flavonoids can act as antioxidants in saline-alkali stress. Higher levels of flavones, flavonols, and isoflavones were found under saline stress. This result supports our results (1). However, the accumulation of flavonoids in the peanut roots was reduced by adding exogenous calcium to the salt stress treatment. We hypothesize that upon receiving a signal of salt stress from the external environment, the addition of exogenous calcium prompts Ca^2+^ to penetrate the cell through Ca^2+^ channels on the cell membrane. This influx elevates the intracellular Ca^2+^ concentration, enabling the plant to respond to salt stress by modulating various physiological and biochemical processes (34). Specifically, the antioxidant enzymes within the plant’s defense system were fortified, and there was an augmentation in the ascorbate acid-glutathione cycle (AsA-GSH cycle). This occurred when flavonoids ceased to be required to furnish additional hydroxyl groups for scavenging excessive ROS, thereby mitigating the detrimental effects of oxidative stress on the plant roots (21, 29). This result supports our hypothesis.

The antioxidant content, comprising total phenols and total flavonoids, along with the antioxidant capacity measured by ABTS assay, exhibited significant increments in peanut roots following 48 h of NaCl treatment (Figure 3). However, a contrasting trend was observed in the DPPH free radical scavenging capacity compared to the ABTS results. This apparent paradox can be elucidated by considering that the surge in total flavonoids and total phenols content might be attributed to an elevation in specific monomeric flavonoids and phenolic acids (Figure 2). The biosynthesis of these compounds is intricately influenced by various enzymes within the phenylpropanoid biosynthesis pathway, three such as PAL, C4H, and 4CL. In addition, flavonoids and phenolics are recognized as compounds that alleviate oxidative stress, and salt stress may initiate the plant’s protective mechanisms, producing more flavonoids and phenolics (29, 35). At the same time, we speculated that Ca^2+^ may act as secondary signals, explicitly enhancing the scavenging ability of certain free radicals (36). This implies that the fluctuations observed in DPPH and ABTS readings may primarily stem from variations in distinct antioxidant compounds.

The quantification of five monomeric flavonoids showed that, except for rutin, the levels of the remaining four monomeric flavonoids were notably elevated under salt treatment compared to Na_Ca treatment. This difference was particularly pronounced during the later stages of cultivation, aligning seamlessly with the overall trend observed in the subclasses of flavonoids. We hypothesized that although the trend of rutin was not consistent with that of flavonoids, the content of flavonoid subclasses was affected by the total accumulation of various monomeric flavonoids. The contents of daidzein, liquiritigenin, and formononetin reached the maximum at 48 h after treatment, and the contents of hyperoside and rutin were maximal at 60 h and 72 h after treatment, respectively, which may be attributed to the accumulation effect of the metabolites. The enzymatic activities crucial to the flavonoid biosynthesis pathway exhibited heightened levels in peanut roots following 48 h of Na treatment compared to Na_Ca treatment. This trend closely mirrored the patterns observed in the five monomeric and total flavonoids (Figures 2, 3). Given the intricate and multifaceted nature of flavonoid biosynthesis, we contend that the levels of monomeric flavonoids are intricately governed by the activity of critical enzymes and the expression of essential genes within the biosynthetic pathway (4). Similarly, the accumulation effects were also reported in peanut sprouts (3).

However, our prior studies established that the optimal timeframe for observing the impact of exogenous calcium on peanut roots under salt stress is after 48 h of cultivation (29, 37). Hence, at the 48th hour post-treatment, we assessed the transcription of eight pivotal enzyme genes involved in the flavonoid biosynthesis pathway via qRT-PCR. Remarkably, these key enzyme genes’ alterations in transcriptional levels and enzyme activities unfolded in tandem after the 48 h mark. Notably, the expression profiles of *AhCHI1* and *AhCHI2* mirrored the pattern observed in CHI activity, *AhIFS1* and *AhIFS2* showed the same trend as IFS activity, and only *AhUGT4* showed the same trend as UGT activity. Moreover, the highest gene transcription appeared in salt treatment, so we can assume that the transcription of genes and protein translation are simultaneous. The expression pattern of *AhUGT1*, *AhUGT2* and *AhUGT3* differed from the trend of UGT activity, which may be due to the different roles of different genes in UGT enzyme synthesis in many gene families. This is the same as reported in wheat and peanut sprouts (3, 38).

This study comprehensively identified 1,037 phytochemicals, encompassing primary and secondary metabolites in peanut roots. This extensive identification surpasses previous reports, establishing a robust foundation for understanding the metabolomic landscape of peanut roots. The exhaustive analysis of the absolute quantitative flavonoid metabolome unveiled that salt stress triggered a notable increase in flavonoid production within peanut roots compared to the control conditions. Interestingly, the sole introduction of exogenous calcium failed to exert a discernible impact on flavonoid accumulation. Nevertheless, introducing exogenous calcium amidst salt stress notably curtailed the accumulation of flavonoids within the roots. This outcome was consistently observed when assessing five monomeric flavonoids at various intervals. In summary, peanut roots exhibited resilience to the detrimental impacts of abiotic stress by upregulating flavonoid production. Additionally, the supplementation of exogenous calcium spurred the activation of residual antioxidant defense mechanisms within the roots, consequently diminishing the allocation of carbon sources to secondary metabolic pathways. Furthermore, exogenous calcium exerted a down-regulatory effect on the activity of pivotal enzymes in the flavonoid biosynthesis pathway and the expression of crucial enzyme genes, ultimately impeding the accumulation of flavonoids. This study offers a fresh outlook on leveraging the full potential of flavonoids in forthcoming endeavours.

## Data Availability

The original contributions presented in the study are publicly available. This data can be found here: https://doi.org/10.6084/m9.figshare.27003640.v1.
