# Supplementary material for: Effects of exogenous calcium on flavonoid biosynthesis and accumulation in peanut roots under salt stress through multi-omics
Source: Front Nutr. 2024 Oct 30;11:1434170. doi: 10.3389/fnut.2024.1434170 (PMC11557398; doi:10.3389/fnut.2024.1434170)
Supplement: Supplementary file 5 [file Image_1.pdf]

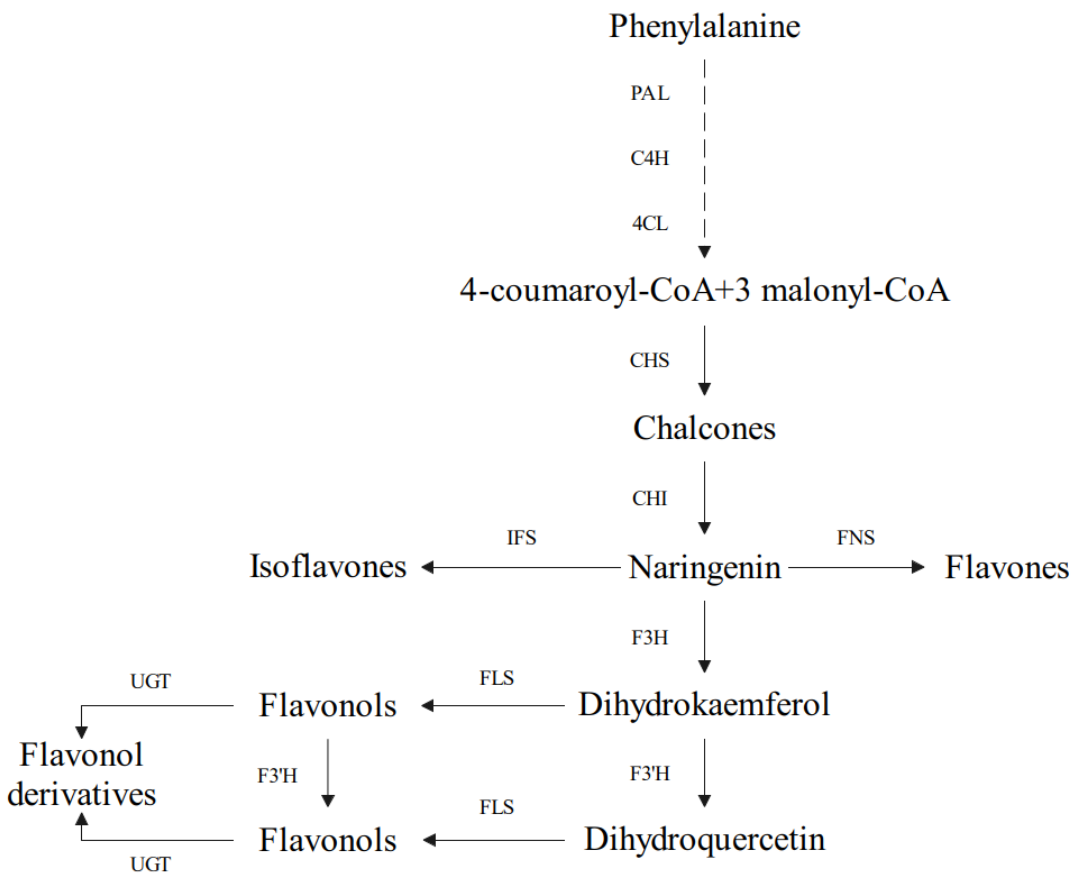

Figure S1 Metabolic pathway of flavonoids in plant. PAL, Phenylalnineammonialyase; C4H, Cinnamate-4-hydroxylase; 4CL, 4-coumarinyl-CoA ligase; CHS, Chalcone synthase; CHI, Chalcone isomerase; IFS, Isoflavone synthase; FNS, Flavone synthase; F3H, Flavanone 3-hydroxylase; F3'H, Flavonoid 3'-hydroxylase; FLS; Flavonol synthase; UGT, UDP-Glycosyltransferase.
